# Retrofractamide C Derived from *Piper longum* Alleviates Xylene-Induced Mouse Ear Edema and Inhibits Phosphorylation of ERK and NF-κB in LPS-Induced J774A.1

**DOI:** 10.3390/molecules25184058

**Published:** 2020-09-05

**Authors:** Hyung Jin Lim, Seon Gyeong Bak, Eun Jae Park, Sae-Kwang Ku, Soyoung Lee, Seung Woong Lee, Kang Min Lee, Seung-Jae Lee, Mun-Chual Rho

**Affiliations:** 1 Immunoregulatory Material Research Center, Korea Research Institute of Bioscience and Biotechnology, Jeongeup-si, Jeonbuk 56212, Korea; lhjin@kribb.re.kr (H.J.L.); tsk9096@kribb.re.kr (S.G.B.); pej911029@kribb.re.kr (E.J.P.); sylee@kribb.re.kr (S.L.); lswdoc@kribb.re.kr (S.W.L.); 2 Department of Molecular Biology, Chonbuk National University, Jeonju-si, Jeonbuk 54896, Korea; kmlee@jbnu.ac.kr; 3 Department of Marine Bio Food Science, Chonnam National University, Yeosu-si, Jeonnam 59626, Korea; 4 Division of Biotechnology and Advanced Institute of Environment and Bioscience, Jeonbuk National University, Iksan-si, Jeonbuk 54596, Korea; 5 Department of Anatomy and Histology, College of Korean Medicine, Daegu Haany University, Gyeongsan-si, Gyeonbuk 38610, Korea; gucci200@hanmail.net

**Keywords:** *Piper longum*, retrofractamide C, xylene-induced ear edema, anti-inflammation

## Abstract

Many studies have reported the biological activities of retrofractamide C (RAC). However, few studies have investigated the anti-inflammatory effect of RAC. In the present study, we investigated the anti-inflammatory effect of RAC using lipopolysaccharide (LPS)-induced J774A.1 cells and a xylene-induced mouse ear edema model. Treatment with RAC decreased LPS-induced nitric oxide (NO) and prostaglandin E2 (PGE2) secretion and inducible NO synthase (iNOS) and cyclooxygenase 2 (COX2) protein expression. It also downregulated the LPS-induced production of interleukin-1β (IL-1β) and interleukin-6 (IL-6) but not tumor necrosis factor α (TNF-α). In the LPS-induced signaling pathway, RAC inhibited the phosphorylation of extracellular signal-regulated kinase (ERK) and nuclear factor kappa light chain enhancer of activated B cells (NF-κB) but not c-Jun N-terminal kinase (JNK) or p38. In a xylene-induced mouse ear edema model, RAC treatment alleviated edema formation and inflammatory cell infiltration. In conclusion, the present study indicates that RAC has the potential to have anti-inflammatory effects and could be a prospective functional food.

## 1. Introduction

The role of the inflammatory response is to defend the body against infection and tissue injury [1,2,3]. The inflammatory response is initiated by recognition of pathogen-associated molecular patterns (PAMPs) of foreign substances or damage-associated molecular patterns (DAMPs) of injured tissue by pattern recognition receptors (PRRs) of residing or circulating immune cells, such as macrophages, mast cells, fibroblasts and leukocytes [3,4]. Toll-like receptor (TLR) family members are PRRs that detect various molecules, such as extracellular components and nucleic acids of bacteria and viruses, according to their subtype [5,6]. Once a PRR binds its agonist, downstream signaling pathways, including mitogen-activated protein kinases (MAPKs) and the nuclear factor kappa light chain enhancer of activated B cells (NF-κB) signaling pathway, are activated. Subsequently, activator protein-1 (AP-1), which is activated by MAPKs, and NF-κB upregulate proinflammatory gene expression [5,6,7,8]. The inflammatory response is mediated and maintained by upregulated proinflammatory cytokines and chemokines. The response induces immune cells to remove pathogenic factors but is often accompanied by pain, vasodilation and fever [9,10]. Thus, acute and chronic inflammatory responses can cause inflammatory diseases, such as sepsis, ulcerative colitis, rheumatoid arthritis and asthma [11,12,13,14].

There are various models to evaluate the anti-inflammatory activities of natural products. The LPS-induced macrophages are commonly used as an in vitro model to access anti-inflammatory activity. LPS stimulated macrophages to produce proinflammatory cytokines and chemokines such as IL-1β, IL-6, IL-8 and monocyte chemoattractant protein 1 (MCP-1) [15]. The severity of inflammation is evaluated by proinflammatory mediators and an anti-inflammatory mechanism is accessed through LPS-induced signaling pathways such as MAPKs and NF-κB. Xylene-induced mouse ear edema model is a simple and widely used animal model for accessing the anti-inflammatory activity of natural products. ICR mouse, which is used in this study, is an albino outbred stain and has been widely used in various research fields, such as pharmacology, toxicology and oncology. It shows an inflammatory response against various stimuli like xylene, formalin, carrageenan and LPS [16]. Xylene application on skin results in an increase in oxidative species and DNA fragmentation [17,18]. It induces iNOS and TNF-α in the skin. Xylene also causes a histopathological change in the skin. For example, swelling, immune cell infiltration and stratum corneum destruction are induced [19].

*Piper longum* L., also called long pepper, is widely cultivated in the tropical and subtropical regions of the world [20,21]. The fruits of *Piper longum* are commonly used as spices and traditional medicines to treat chronic bronchitis, cough, cold and poisonous snake bites [22]. Various biological properties, such as antioxidative, insecticidal, antimicrobial, antitumor and anti-inflammatory properties, have been reported [23,24,25]. The phytochemical constituents of fruits of *Piper longum* are known to be terpenes, lignans, flavones and alkaloids [26]. In a previous study, we isolated three alkamides, including retrofractamide C (RAC), from fruits of *Piper longum* and reported the inhibitory effect on diacylglycerol acyltransferase and cell adhesion [27,28]. It is also reported that RAC activates transient receptor potential vanilloid 1 (TRPV1), which is associated with anti-inflammation and anticancer [29]. However, the anti-inflammatory activity of RAC has been poorly studied.

In this study, we investigated the anti-inflammatory activity of RAC on LPS-induced J774A.1 cells and its therapeutic effect against xylene-induced mouse ear edema acute inflammation model.

## 2. Results and Discussion

### 2.1. RAC Inhibits NO and PGE2 Production in LPS-Induced J774A.1 Cells

**Figure 1:**
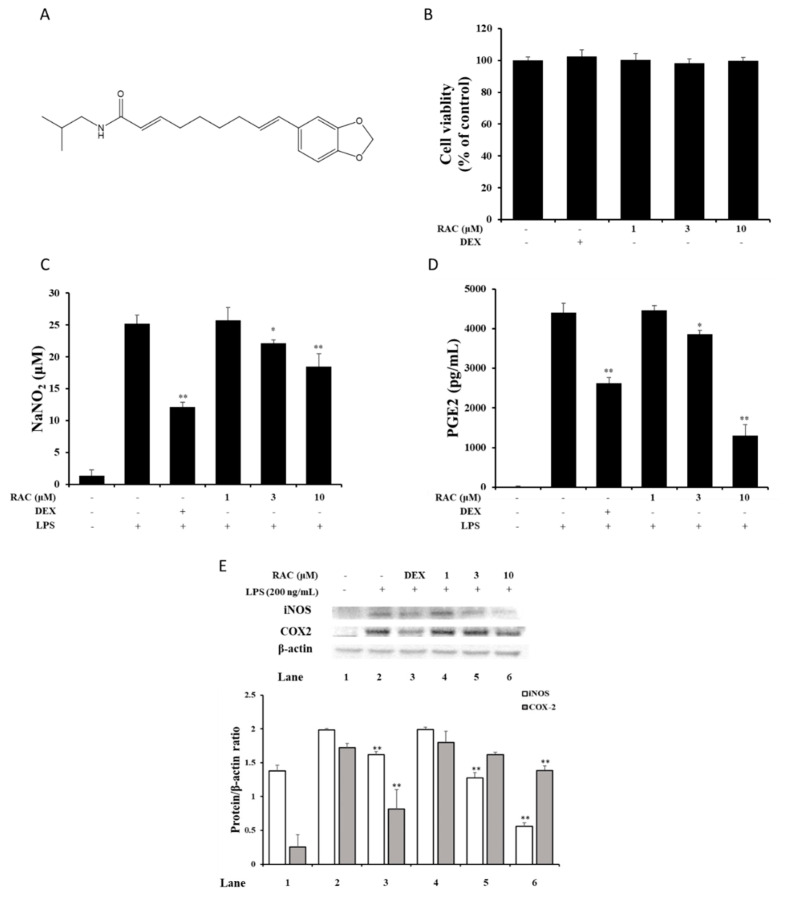
Inhibitory effect of retrofractamide C (RAC) on lipopolysaccharide (LPS)-induced nitric oxide (NO) and PGE2 production and iNOS and COX2 protein expression in J774A.1 cells. (**A**) Chemical structure of retrofractamide C (RAC). (**B**) Cytotoxicity of RAC. J774A.1 cells were treated with the indicated concentrations of dexamethasone and RAC for 24 h. Cytotoxicity was determined by MTT assay. (**C**,**D**) Inhibition of NO and PGE2 production by RAC. J774A.1 cells were pretreated with 10 μM dexamethasone or 1, 3 or 10 μM RAC for 1 h before treatment with 200 ng/mL LPS for 18 h. (**C**) The secretion of NO was measured by an NO assay. (**D**) PGE2 production was determined by ELISA. (**E**) iNOS and COX2 protein expression decreased by RAC treatment. J774A.1 cells were treated with 200 ng/mL LPS for 18 h after treatment with dexamethasone and RAC for 1 h. The iNOS and COX2 protein expression levels were determined by immunoblot assay. The band optical densities were calculated by ImageJ software. Representative data are presented. Values are presented as the mean ± SD of three individual experiments. * *p* < 0.05, ** *p* < 0.01 compared with the LPS only-treated group. DEX, 10 μM dexamethasone.

NO and PGE2 are major mediators of the inflammatory response. Their biosynthesis is significantly upregulated in inflamed regions, and they contribute to the pathogenesis of inflammatory disorders [30,31]. NO is produced by NO synthase (NOS) and converted from L-arginine in the presence of NADPH and oxygen. In this process, tetrahydrobiopterin (BH4), flavin adenine dinucleotide (FAD), flavin mononucleotide (FMN) and heme act as a cofactor [32]. PGE2 synthesis is initiated with arachidonic acid formation from phospholipids of the cell membrane by phospholipase A2 (PLA2). Arachidonic acid is converted to prostaglandin H 2 (PGH2) by cyclooxygenase (COX) then, PGH2 is converted to PGE2 by PGE2 synthase [33]. NO and PGE productions are regulated by substrates availability, enzyme expressions and enzyme activities. Previous studies showed that reducing NO and PGE2 levels could reduce the symptoms of inflammatory diseases [30,31,32,33,34]. Before investigating the anti-inflammatory effect of RAC (Figure 1A), an MTT assay for the cytotoxicity of RAC was performed. There was no cytotoxicity on J774A.1 cells at the indicated concentration of RAC (Figure 1B). After that, we investigated the effect of RAC on LPS-induced NO and PGE2 production to evaluate the anti-inflammatory effect of RAC. RAC treatment inhibited both NO and PGE2 production in a dose-dependent manner (Figure 1C, D).

### 2.2. RAC Decreases iNOS and COX2 Expression in LPS-Induced J774A.1 Cells

The synthesis of NO and PGE2 is achieved by NOS and COX enzymes. They have several isotype enzymes; however, specific enzymes, iNOS and COX2, are responsible for NO and PGE2 production in the inflammatory response [30,31,32]. Inhibition of their activity and expression reduces NO and PGE2 levels and could alleviate inflammatory disorders [31,32]. To determine whether RAC affected LPS-induced iNOS and COX2 protein expressions, immunoblot assay was performed. iNOS expression was significantly decreased after 3 and 10 μM RAC treatment, and COX2 expression was significantly downregulated after 10 μM RAC treatment (Figure 1E). There are several factors, which regulated both iNOS and COX2 expressions, including transforming growth factor β (TGF-β), NF-κB and AP-1. In macrophage cells, TGF-β inhibits COX2 expression and it increases degradation of iNOS and decreases iNOS mRNA stability [32,33]. The expressions of iNOS and COX2 are also regulated by NF- κB and AP-1, which are transcriptional factors. Thus, RAC treatment could affect these factors and downregulation of iNOS and COX2 protein expressions.

### 2.3. RAC Inhibits IL-1β and IL-6 But Not TNF-α Gene Expression in LPS-Induced J774A.1 Cells

**Figure 2:**
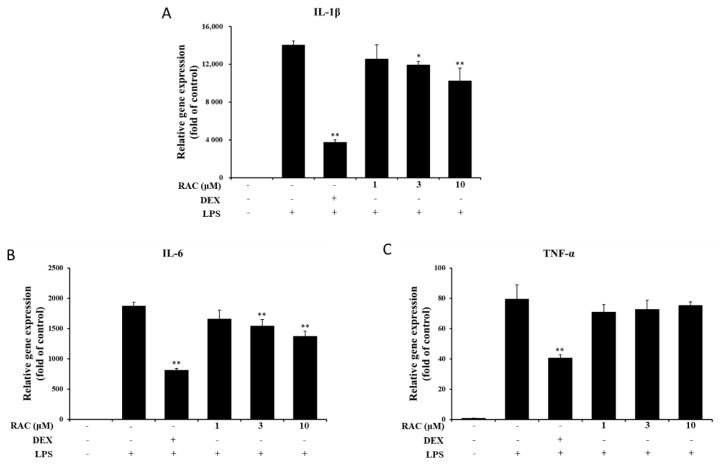
Effect of RAC on LPS-induced proinflammatory gene expression. J774A.1 cells were treated with 200 ng/mL LPS after pretreatment with 10 μM dexamethasone or 1, 3 or 10 μM RAC for 1 h. (**A**) IL-1β, (**B**) IL-6 and (**C**) TNF-α gene expression was determined by quantitative real-time PCR. The data were normalized to 18S rRNA expression and are presented as the fold change compared to the untreated group. The results are presented as the mean ± SD of three individual experiments. * *p* < 0.05, ** *p* < 0.01 compared with the LPS only-treated group. DEX, 10 μM dexamethasone.

Proinflammatory cytokines induced by activated innate immune cells mediate and amplify the inflammatory response through activation of their downstream signaling cascades [34,35]. They are also related to the pathogenesis and hyperalgesia of inflammatory disorders [36,37,38]. The effect of RAC on proinflammatory cytokine expression was evaluated by quantitative real-time PCR. As shown, LPS-induced IL-1β and IL-6 expression was significantly decreased after 3 and 10 μM RAC treatment, but TNF-α expression was not affected (Figure 2). Regulation of these cytokine gene expressions is affected by various factors. LPS stimulation leads to activation of transcription factors such as NF-κB, AP-1, IRF and CCAAT/enhancer-binding protein β (C/EBPβ) and they promote proinflammatory cytokine gene expressions [39]. The cytokine mRNA is controlled by post-transcriptional regulation. Binding of RNA binding proteins or microRNA with cytokine mRNA decreases RNA stability and increases mRNA degradations [40].

### 2.4. RAC Decreases Phosphorylated ERK and NF-κB p65 But Not JNK or p38 in LPS-Induced J774A.1 Cells

**Figure 3:**
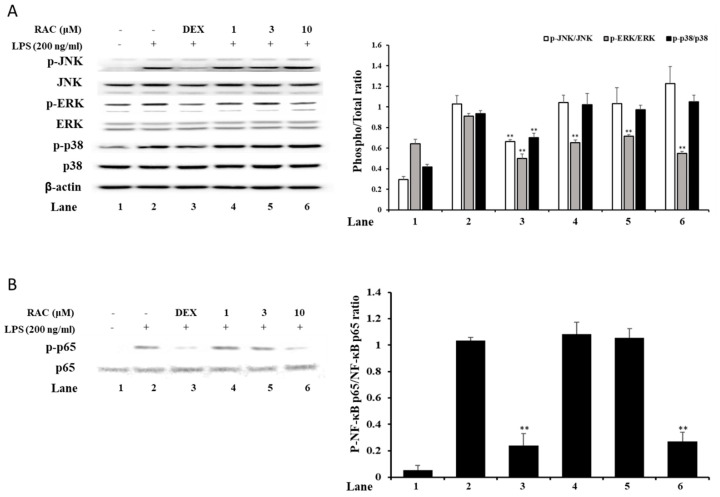
Effect of RAC on LPS-induced MAPKs and the NF-κB signaling pathway. J774A.1 cells were pretreated with 10 μM dexamethasone or 1, 3 or 10 μM RAC for 1 h before treatment with 200 ng/mL LPS for 30 min to 1 h. (**A**) The protein expression of MAPKs was determined by immunoblot. Band optical densities were calculated by ImageJ software. (**B**) NF-κB p65 subunit expression was measured by immunoblot analysis, and the band optical density was calculated by ImageJ software. The results are representative of three independent experiments. Values are presented as the mean ± SD of three individual experiments. * *p* < 0.05, ** *p* < 0.01 compared with the LPS only-treated group.

MAPKs and NF-κB signaling are involved in the TLR family signaling pathway, as part of the downstream signaling cascade [5,6]. Inhibition of these signaling molecules is a good strategy to treat inflammatory diseases. To investigate the effect of RAC on MAPKs and NF-κB signaling molecules, immunoblot analysis was performed. The results showed that RAC treatment downregulated the phosphorylation of ERK and NF-κB p65 (Figure 3A,B). However, it did not affect the phosphorylation of JNK and p38 (Figure 3A). These results indicate that RAC selectively regulates signaling molecules, ERK and NF-κB. There are some studies showing that selective inhibition of ERK and NF-κB could not affect the production of TNF-α but decreased other inflammatory mediators, such as IL-1β, IL-6, iNOS and COX2 [41,42,43]. It has also been reported that selective inhibition of p38 or JNK downregulated TNF-α as well as other inflammatory mediators [44]. These results imply that p38 and JNK MAPK signaling are deeply involved in TNF-α expression.

### 2.5. RAC Alleviates Xylene-Induced Mouse Ear Edema

**Figure 4:**
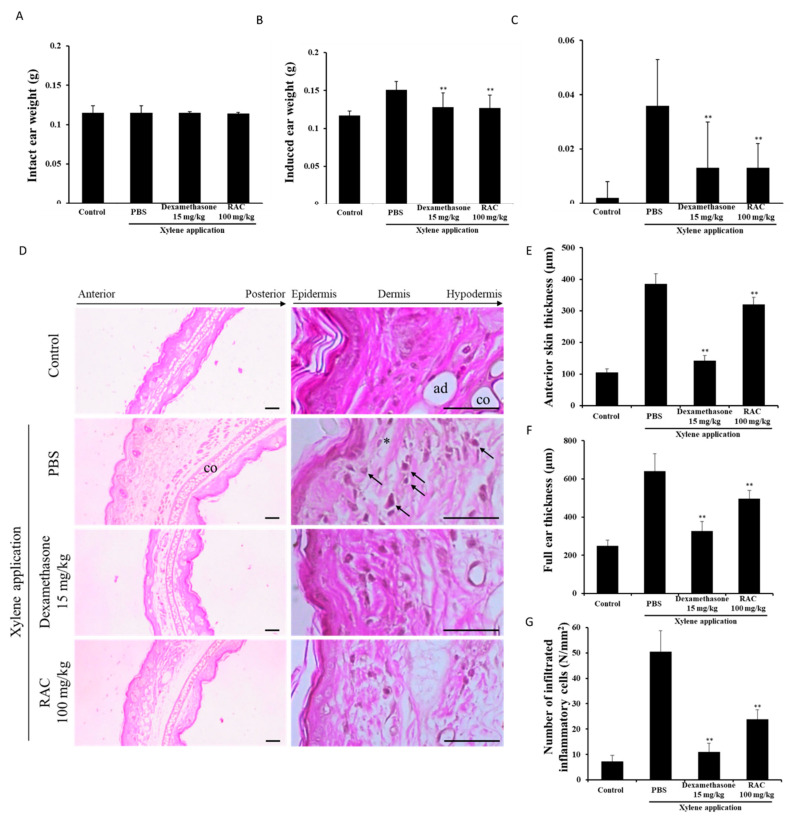
Effect of RAC on the xylene-induced mouse ear edema model. The mice were pretreated with PBS or dexamethasone (15 mg/kg) intraperitoneally or RAC (100 mg/kg) orally before xylene application on the anterior surface of the right ear for 2 h. (**A**) Intact ear weight, (**B**) induced ear weight and (**C**) differences in ear weight were measured. (**D**–**G**) For histological analysis, mouse ears were fixed and stained with H&E. (**D**) Representative H&E-stained mouse ear images of each group. Severe vasodilation (asterisks) and infiltration of inflammatory cells (arrows) were observed in the xylene only-treated group. (**E**) Anterior skin thickness, (**F**) full ear thickness and (**G**) the number of infiltrated inflammatory cells were measured. Values are presented as the mean ± SD of nine experimental individuals. * *p* < 0.05, ** *p* < 0.01 compared with the xylene-only-treated group. ad, adipocytes; co, chondrocytes. Scale bars represent 80 μm.

*Xylene-*induced ear edema is a simple and classic model for acute inflammation studies. It is widely used to evaluate the anti-inflammatory activities of substances [16]. In this study, we assessed the anti-inflammatory effect of RAC on a xylene-induced ear edema model. The administration of PBS, dexamethasone and RAC did not affect intact ear weights (Figure 4A). The xylene application induced ear weight and differences in ear weight, but dexamethasone and RAC significantly decreased them compared with those of the xylene-only-treated group (Figure 4B,C).

Acute inflammation is initiated by the recognition of harmful stimuli by immune cells [1,2]. Immune cells, such as macrophages and mastocytes, release proinflammatory mediators like NO, PGE2, histamine, proinflammatory cytokines and chemokines. Histamine, NO and PGE2 cause vasodilation and increased vascular permeability through relaxing smooth muscle [31,45]. Proinflammatory cytokines and chemokines induce cell adhesion molecules, such as ICAM-1 and VCAM-1, recruit immune cells like neutrophils and leukocytes through chemotaxis [46]. These responses cases immune cell infiltration and edema formation. In a histological analysis of the induced ear, xylene application induced severe vasodilation, edema formation, and immune cell infiltration. The dexamethasone and RAC treatments significantly reduced these factors and skin thickness compared with those of the xylene-only-treated groups (Figure 4D–G). These results may be due to RAC-downregulated inflammatory mediators, which promote vasodilation and increase vascular permeability.

## 3. Materials and Methods

### 3.1. Materials and Reagents

J774A.1 cells were purchased from the American Type Culture Collection (ATCC; Rockville, MD, USA). The cells were cultured in DMEM (Gibco BRL; Grand Island, NY, USA) supplemented with 10% fetal bovine serum (FBS), 50 U/mL penicillin and 50 mg/mL streptomycin at 37 °C in a 5% CO_2_ incubator. Thiazolyl blue tetrazolium bromide, Griess reagent and dexamethasone were obtained from Sigma Aldrich (St. Louis, MO, USA). All antibodies for immunoblot analysis were obtained from Cell Signaling Technology (Danvers, MA, USA).

### 3.2. Isolation of Retrofractamide C

Retrofractamide C was purified from the fruits of *Piper longum* as previously described [22,23]. In brief, dried fruits of *Piper longum* (100 g) were purchased at an herbal marker (Daejeon, Korea). The authenticity of the plant was confirmed by Prof. K. H. Bae, College of Pharmacy, Chungnam National University, Daejeon, Korea. Voucher specimens (PBC-413A and PBC-441A) were deposited in the Korea Plant Extract Bank, Korea Research Institute of Bioscience and Biotechnology. The dried fruits were extracted with methanol (MeOH, 1 L) for 7 days at room temperature. The MeOH extract was evaporated *in vacuo* to yield a residue (32 g). The residue was suspended in distilled water and extracted with chloroform. The chloroform-soluble fraction (7.5 g) was loaded onto a silica gel (230–400 mesh, 1 kg, Merck, Darmstadt, Germany) column for chromatography and eluted with a stepwise gradient of increasing concentrations of chloroform/MeOH (100:0, 80:1, 60:1, 40:1, 20:1, 10:1, 5:1, and 1:1, each 3 L, v/v) to obtain eight subfractions (PLM1–8) based on thin-layer chromatography (TLC) profiles. PLM1 was subjected to silica gel (230–400 mesh, 400 g, Merck) column chromatography and fractionated with hexane/ethylacetate (EtOAc) (50:1, 20:1, 10:1, 7:1, 5:1, 3:1, and 1:1, each 2 L, v/v) to generate eight subfractions (PLM1A-H). PLM1A was separated by semipreparative HPLC (YMC J’sphere ODS-H80 column, 4 μm, 250 × 20 mm, flow rate 4 mL/min) using isocratic elution with 80% MeOH in water to yield RAC (29.9 mg, retention time (tR) 39 min). The semipreparative HPLC consisted of a Shimadzu LC-6AD pump (Shimadzu, Tokyo, Japan) equipped with an SPD-10A detector (Shimadzu) using a YMC J’sphere ODS-H80 column (YMC, Kyoto, Japan).

### 3.3. MTT Assay

J774A.1 cells were seeded in 96-well plates, treated with 1, 3 and 10 μM of RAC for 24 h and treated with thiazolyl blue tetrazolium bromide for 3 h. After incubation, the supernatant was removed, and the remaining formazan was dissolved in dimethyl sulfoxide (DMSO). The absorbance was measured at 540 nm using a microplate ELISA reader (Molecular Devices, Sunnyvale, CA, USA).

### 3.4. NO Assay

J774A.1 cells were cultured in 96-well plates and treated with 200 ng/mL LPS for 18 h after pretreatment with dexamethasone and RAC for 1 h. Then, the supernatant was collected and treated with Griess reagent. The absorbance was measured at 540 nm using a microplate ELISA reader.

### 3.5. ELISA

J774A.1 cells were seeded in 6-well plates and treated with 200 ng/mL LPS for 18 h after pretreatment with dexamethasone and RAC for 1 h. Then, the supernatant was collected, and the prostaglandin E2 (PGE2) concentration was measured by a mouse PGE2 ELISA kit (R&D Systems, Minneapolis, MN, USA) following the manufacturer’s instructions. The absorbance was measured at 450 nm using a microplate ELISA reader.

### 3.6. Quantitative Real-Time PCR

J774A.1 cells were seeded in 6-well plates, and cells were pretreated with the indicated concentrations of dexamethasone and RAC and then treated with 200 ng/mL LPS for 12 h. Total RNA was extracted by a PureLink RNA Mini Kit (Invitrogen, San Diego, CA, USA) following the manufacturer’s instructions. Complementary DNA (cDNA) was synthesized using a PrimeScript 1st strand cDNA synthesis kit (Takara Bio Inc., Shiga, Japan) and subjected to quantitative real-time PCR. Quantitative real-time PCR was performed by a StepOnePlus Real-Time PCR S machine using a TaqMan probe and TaqMan PCR master mix (Applied Biosystems, Foster City, CA, USA).

### 3.7. Immunoblot Analysis

J774A.1 cells were seeded in 6-well plates and treated with 200 ng/mL LPS for the indicated times after pretreatment with dexamethasone and RAC for 1 h. Total proteins were extracted using cell lysis buffer supplemented with phosphatase and proteinase cocktail (Cell Signaling Technology). The total protein concentration was measured, and equal amounts of protein were subjected to 4–12% SDS-PAGE. Separated proteins were transferred onto polyvinylidene fluoride (PVDF) membranes and blocked with tris-buffered saline (TBS) containing 5% skim milk. After blocking, the membrane was washed with TBS containing 0.1% Tween-20 (TBST) and incubated with the appropriate primary and secondary antibodies. Finally, the membrane was developed using a West-Queen RTS Western Blot Detection Kit (iNtRON Bio., Seongnam, Korea).

### 3.8. Animals and Induction of a Xylene-Induced Ear Edema Model

Detail procedures were described in a previous study [47]. In brief, six-week-old male ICR mice were purchased from OrientBio (Kwangju, Korea) and randomly divided into 4 groups (*n* = 9 mice per group): PBS intraperitoneally injected and administered mice (Intact control), PBS intraperitoneally injected and xylene-administered mice (Xylene control), dexamethasone intraperitoneally injected and xylene-administered mice, and RAC orally administered and xylene-administered mice. Before 30 min of topical application of PBS or xylene on the anterior surface of the right ear for 2 h, the mice were administered PBS, 15 mg/kg dexamethasone intraperitoneally and 100 mg/kg RAC orally. After the topical application of 0.03 mL of PBS and xylene on the anterior surface of the right ear, the mice were sacrificed, and changes in ear weight and histopathology were measured. For histological analysis, mouse ears were fixed in 10% formalin, embedded in paraffin, sectioned and stained with hematoxylin and eosin (H&E) following the general procedure. The experimental protocols were approved by the Institutional Animal Care and Use Committee of Korea Research Institute of Bioscience and Biotechnology (permission number KRIBB-AEC-17059). All mice were treated according to the Guide for the Care and Use of Laboratory Animals published by the US National Institutes of Health.

### 3.9. Statistical Analysis

The results are presented as the mean ± standard deviation (SD) of three or nine individual experiments. Statistical analysis was performed using Prism 5 software (GraphPad Software, San Diego, CA, USA) for in vitro results and one-way ANOVA followed by Tukey’s test for in vivo results.

## 4. Conclusions

In this study, we evaluated the anti-inflammatory effect of RAC from *Piper longum* through in vitro and in vivo experiments. RAC decreased NO and PGE2 production and the protein expression of their synthesis enzymes. The gene expression of the IL-1β and IL-6 proinflammatory cytokines but not TNF-α expression was inhibited by RAC treatment. Immunoblot analysis of MAPKs and NF-κB signaling molecules showed specific inhibition of ERK and NF-κB p65 phosphorylation by RAC. Finally, RAC treatment alleviated xylene-induced ear edema. Taken together with our previous research on the inhibitory activity on cell adhesion, the present results confirm the anti-inflammatory activity of RAC. Based on our results, this alkamide could be a useful candidate for anti-inflammatory agent development.
